# Author Correction: Limited surface impacts of the January 2021 sudden stratospheric warming

**DOI:** 10.1038/s41467-022-31311-6

**Published:** 2022-06-20

**Authors:** N. A. Davis, J. H. Richter, A. A. Glanville, J. Edwards, E. LaJoie

**Affiliations:** 1 Atmospheric Chemistry Observations and Modeling Laboratory, National Center for Atmospheric Research, Boulder, CO USA; 2 Climate and Global Dynamics Laboratory, National Center for Atmospheric Research, Boulder, CO USA; 3 NOAA/NCEP/Climate Prediction Center, College Park, MD USA

**Keywords:** Atmospheric science, Natural hazards

Correction to: *Nature Communications* 10.1038/s41467-022-28836-1, published online 3 March 2022.

The original version of this Article contained an error in Fig. 5, in which the legend incorrectly states that the magenta/black shading indicates downward/upward wave propagation This has been corrected in both the PDF and HTML versions of the Article.

