# Supplementary material for: Limited surface impacts of the January 2021 sudden stratospheric warming
Source: Nat Commun. 2022 Mar 3;13:1136. doi: 10.1038/s41467-022-28836-1 (PMC8894394; doi:10.1038/s41467-022-28836-1)
Supplement: Supplementary file 1 — Supplementary Information [file 41467_2022_28836_MOESM1_ESM.pdf]

Supplementary Information for "Limited surface impacts of the January 2021 sudden stratospheric warming"

Davis, N. A.[1], Richter, J. H.[2], Glanville, A. A.[2], Edwards, J.[2], LaJoie, E.[3]

[1]*Atmospheric Chemistry Observations and Modeling Laboratory, National Center for Atmospheric Research, Boulder, CO, USA*

[2]*Climate and Global Dynamics Laboratory, National Center for Atmospheric Research, Boulder, CO, USA*

[3]*NOAA/NCEP/Climate Prediction Center, College Park, MD, USA*

SSW occurrence in each CESM2(WACCM6) forecast ensemble

Fig. S1 displays the strength of the stratospheric polar vortex, taken here as the zonal mean zonal wind at 10 hPa and 60°N, in all ensemble members for all four forecasts. A sudden stratospheric warming is diagnosed when the zonal mean zonal wind first becomes negative.

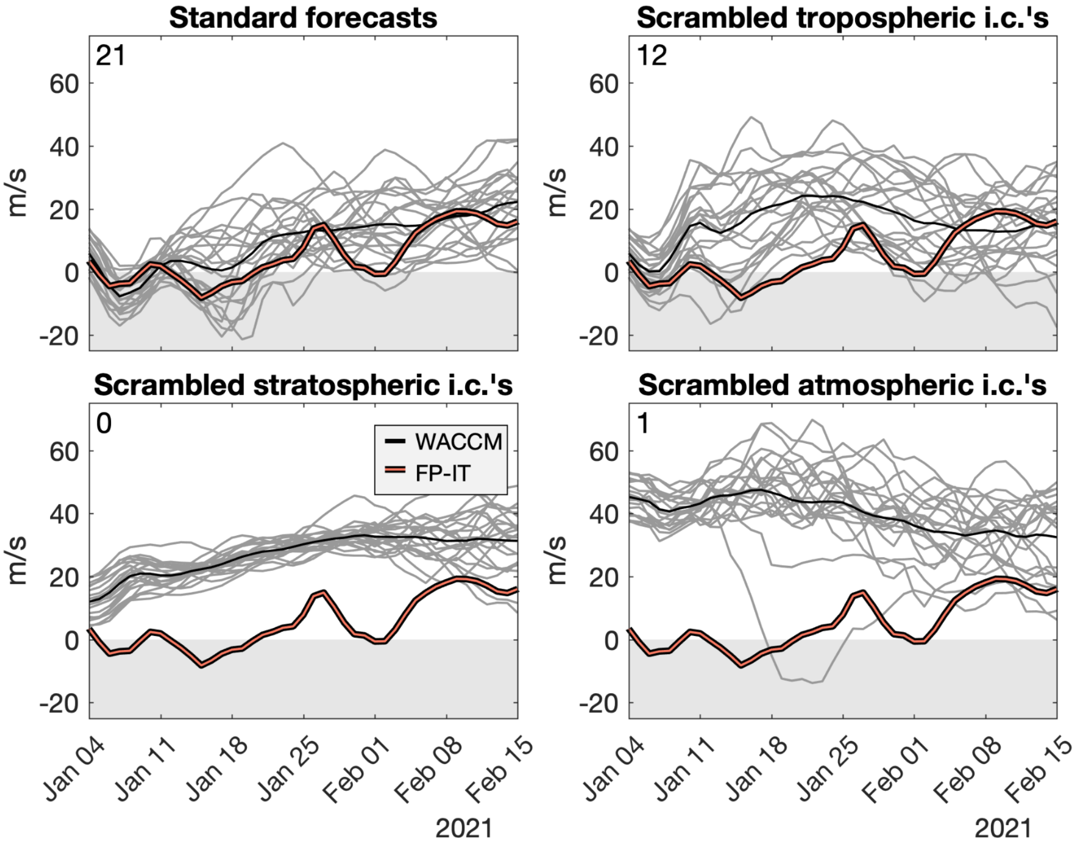

Figure S1: Zonal mean zonal wind at 60 degrees North and 10 hPa in (blue) all CESM2(WACCM6) forecasts and (orange) NASA FP-IT, used here for verification. The number of SSWs encountered in each forecast ensemble is displayed in the upper left.

NOAA SubX week 3-4 surface temperature forecasts

Forecast data for all models participating in NOAA SubX can be accessed at http://cola.gmu.edu/subx/index.html. Fig. S1 displays the week 3-4 surface temperature forecasts from each model, as well as from MERRA2 and the multi-model-mean (MME). Not all models initialize their forecasts on the same date, but all forecasts in Fig. S1 are valid for January 19-February 1. Models and the agency/university that produce their forecasts are listed in Table S1.

Table S1: Agency/university, model, and SubX model name

Agency/university	Model	Model name
Rosenstiel School of Marine and Atmospheric Science, University of Miami (RSMAS)	Community Climate System Model version 4 (CCSM4)	RSMAS CCSM4
National Center for Atmospheric Research (NCAR)	Community Earth System Model version 2, Whole Atmosphere Community Climate Model (CESM2(WACCM6))	NCAR CESM2(WACCM6)
NOAA National Centers for Environmental Prediction (NCEP)	Climate Forecast System version 2 (CFSv2)	NCEP CFSv2
Environment and Climate Change Canada (ECCC)	Global Environmental Multiscale Model (GEM)	ECCC GEM
NOAA Earth System Research Laboratory (ESRL)	Flow-following Icosahedral Model (FIM)	ESRL FIM
NOAA NCEP	Global Ensemble Forecasts System (GEFS)	NCEP GEFS
National Aeronautics and Space Administration (NASA) Global Modeling and Assimilation Office (GMAO)	Goddard Earth Observing System Model, Version 5 (GEOS5)	GMAO GEOS5
United States Navy Naval Research Laboratory (NRL)	Naval Earth System Model (NESM)	NRL NESM

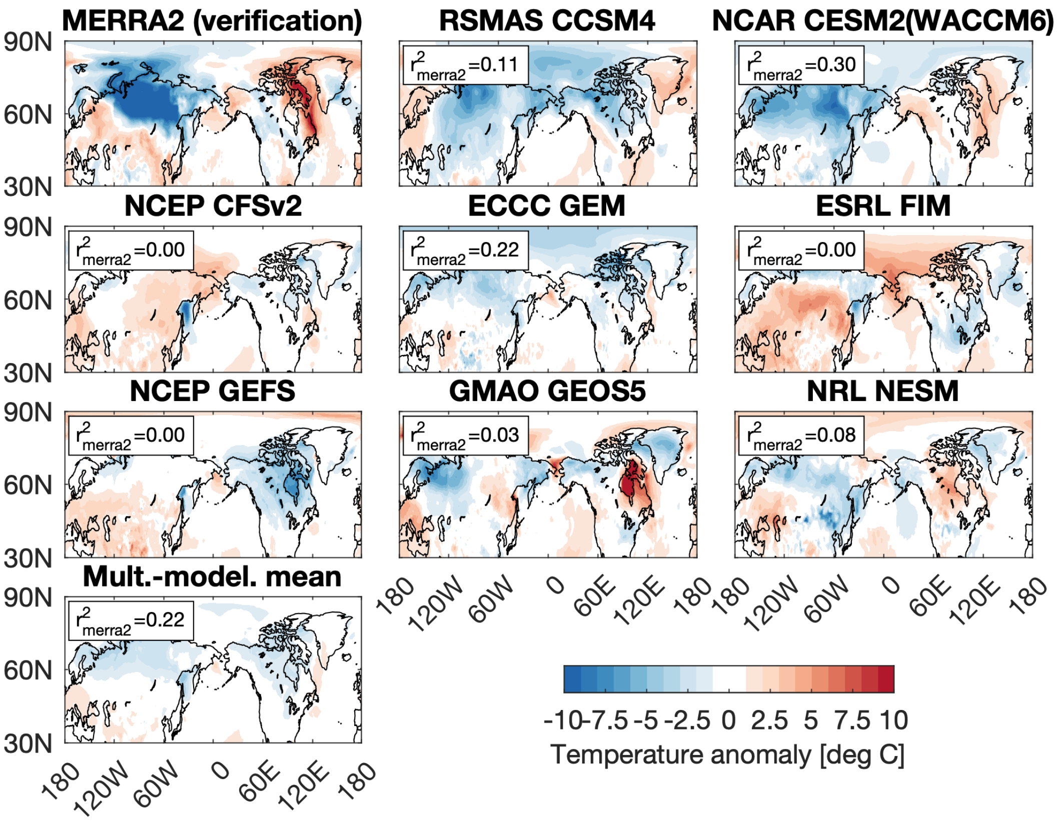

Figure S2: Week 3-4 surface temperature anomalies for MERRA2 and the surface temperature anomaly forecasts from all models participating in NOAA SubX, as well as the multi-model mean. The squared anomaly correlation coefficient between each forecast and MERRA2 is displayed in each panel.

Surface temperature anomalies in MERRA2 and the forecasts initialized on February 8th, 2021

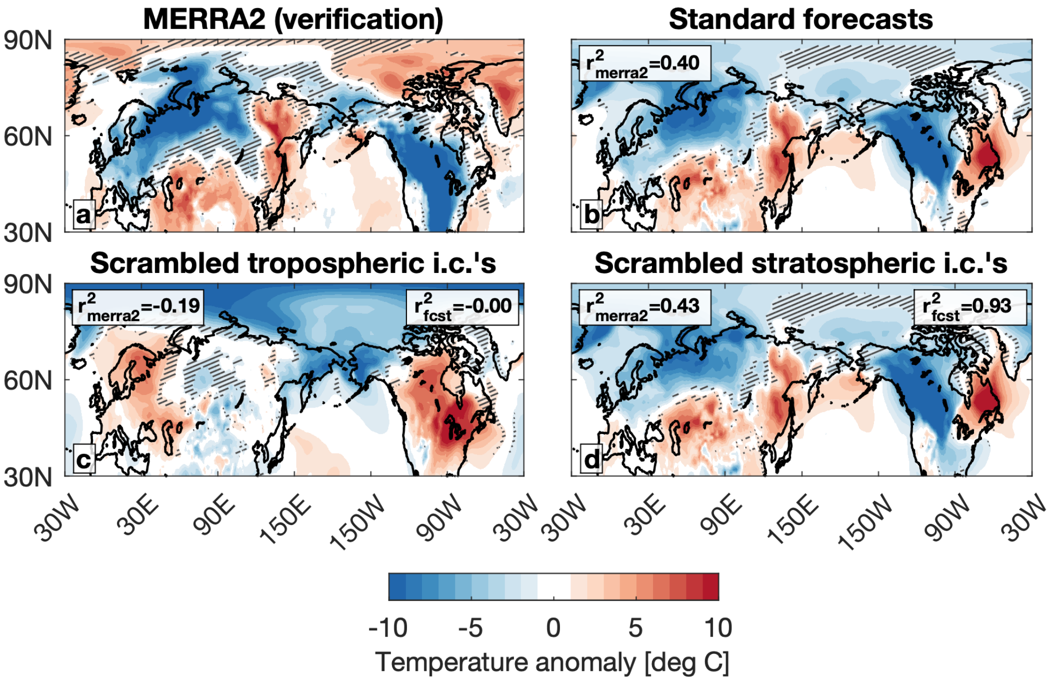

Figure S3: As in Fig. 1, except for the period February 12-18th, 2021 with forecasts initialized on February 8th, 2021.
